# State Medical Examining and Licensing Board

**Published:** 1891-10

**Authors:** 


### STATE MEDICAL EXAMINING AND LICENSING BOARD.

The first meeting of this important body was held in the Lieutenant-Governor's Chamber, in the State Capitol, at Albany, September 1, 1891.

The meeting was called to order by the temporary chairman Dr. W. C. Wey, of Elmira. A ballot having been ordered, Dr. Wm. Warren Potter, of Buffalo, was elected president; Dr. Potter declined the office for personal reasons, whereupon Dr. W. C. Wey was elected President, and M. J. Lewi, of Albany, was elected Secretary. The board then took a recess to attend a conference of the joint boards of medical examiners, and on re-assembling ratified the following recommendations to the Board of Regents:

1. A medical syllabus to be prepared (covering all subjects in which examinations are to be held) under the direction and supervision of a committee of six, to consist of two members appointed from each board.

2. A committee on questions composed of six members, two to be appointed from and by each board, whose duty it shall be on the call of the Secretary of the State Board of Regents, to convene at Albany and revise all questions submitted by the individual examiners under section five of the law.

3. Each question paper on the seven topics shall consist of fifteen questions, of which the candidate shall cancel five, and shall be marked on his answers to the remaining ten.

4. One hundred points shall be the standard, and each candidate must secure seventy-five points in every branch before his or her name shall be recommended to the Regents as a proper person to be licensed to practice medicine in the State.

5. Four examinations shall be held during the coming year, the dates of which may be fixed at the discretion of the Board of Regents.

6. All examinations shall be conducted in the English language.

7. Examinations for medical license shall be conducted in all subjects at one continuous session.

8. No conference of the Boards of Medical Examiners shall be held, excepting on request of a joint call by a majority of the members of at least two of said boards, as certified to the Secretary of the Board of Regents by the proper officers.

9. If, in the opinion of the Board of Regents, it becomes necessary or advisable, additional examinations shall be called as occasion may arise.

10. At least sixty questions shall be sent by registered letter to the Secretary of the Board of Regents, on or before September 15, 1891.

The following assignments of topics were made :

Physiology and Hygiene, Wm. C. Wey; Surgery, George Ryerson Fowler ; Obstetrics, William Warren Potter ; Anatomy, William S. Ely ; Chemistry and Materia Medica, Maurice J. Lewi ; Pathology and Diagnosis, J. P. Creveling ; Theory and Practice and Therapeutics, Eugene Beach.

Drs. Fowler, of Brooklyn, and Lewi, of Albany, were selected as members of the Questions and Syllabus Committee. Dr. Potter moved, and it was carried, that in regard to printed matter the form of heading be submitted to the Secretary of the Board of Regents. Dr. Creveling moved, and it was so ordered, that a circular letter be sent to the members and delegates of the State Medical Society by the Secretary, containing such features as would give them a knowledge of our proceedings and plans. On motion, the Board adjourned subject to the call of its officers.

W. C. WEY, *President.*

MAURICE J. LEWI, *Secretary,*
71 Lancaster St., Albany, N. Y.

